# Comparative Assessment of Female Sexual Function Following Transobturator Midurethral Sling for Stress Urinary Incontinence

**DOI:** 10.3390/ijerph18052286

**Published:** 2021-02-25

**Authors:** Maciej Zalewski, Gabriela Kołodyńska, Agata Zalewska, Waldemar Andrzejewski

**Affiliations:** 1 Department of Gynaecology and Obstetrics, Faculty of Health Sciences, Medical University of Wrocław, 50-367 Wrocław, Poland; zalewskim@interia.pl; 2 Department of Massage and Physical Therapy, Faculty of Physiotherapy, University School of Physical Education, al. I. Paderewskiego 35, 51-612 Wrocław, Poland; waldemar.andrzejewski@awf.wroc.pl; 3 Department of Otolaryngology, Faculty of Medicine and Dentistry, Medical University of Wrocław, 50-367 Wrocław, Poland; agatazalewskaorl@gmail.com

**Keywords:** urinary incontinence, surgical treatment, sexuality

## Abstract

Urinary incontinence (UI) is a significant social problem. According to the World Health Organization, UI affects as much as 30% of premenopausal women and 60% of postmenopausal women. Urinary incontinence can lead to certain problems that negatively affect a woman’s sex life. They result from the fact that certain processes take place in the body during intercourse. As a result of orgasm, the intra-abdominal pressure increases, which in women with urinary incontinence can cause an uncontrolled leakage of urine. The discomfort that this causes, in turn, lowers sexual attractiveness, as well as causes embarrassment. The study involved 50 patients hospitalized in the period from February to May 2019 at the Gynecology Department of the Independent Public Healthcare Center of the Ministry of the Interior and Administration in Wrocław. All patients underwent surgical treatment of stress urinary incontinence using the transobturator tape (TOT) method. To assess sexuality, the international standardized Female Sexual Function Index (FSFI) questionnaire. Analysis of the results obtained from the FSFI questionnaire shows that the operation significantly affects the reduction in pain sensation during intercourse, a reduction in the sensation of sexual arousal, and a worsening vaginal wetness. Stress urinary incontinence significantly affects women’s sex life.

## 1. Introduction

Urinary incontinence (UI) is a significant social problem. According to the World Health Organization, it affects as much as 30% of premenopausal women and 60% of postmenopausal women. Every second person above the age of 70, both women and men, suffer from that condition [1]. However, there are no exact data which could be used to determine a specific number of people affected by urinary incontinence. This is essentially due to the fact that patients often tend not to report their ailments. During the 7th Global Forum on Incontinence in 2018, it was established that the urinary incontinence occurs in 6–10% of the population [2]. It is estimated that the problem affects approximately 424 million people worldwide—303 million women and 121 million men. Incontinence episodes are experienced by approximately 10% of the adult female population. Approximately 30–40% of females in the premenopausal stage and 60% of those in the postmenopausal stage are affected by the condition [3].

The patients suffering from stress urinary incontinence (SUI) should be first subjected to conservative treatment, which includes physical therapy, pharmacotherapy, and behavioral therapy. If those methods turn out to be ineffective, surgical treatment shall be applied. The surgical treatment of SUI often involves the use of slings: Tension-free vaginal tape (TVT) or transobturator tape (TOT), which are placed under the middle section of the urethra. The effectiveness of these treatments is estimated at over 90%. According to WHO, however, the improvement in physical well-being alone is not sufficient for full therapeutic success, the progress in the social and mental sphere is also important. The latter includes such aspects as the lack of pain symptoms or the sexual life satisfaction after surgery [4].

Sexuality has always aroused a large amount of controversy in society. Usually, it is a taboo subject that is not discussed with family, friends, or doctors [5,6,7]. In the biological aspect, sexuality is associated with experiencing physical pleasure, satisfying sexual needs, and relieving sexual tension. However, it is a mistake to analyze sexuality in its biological aspect only [8]. The psychological and social dimensions also play a great role. The sexual sphere cannot be separated from emotions, feelings, excitement, and interest in one’s own body or that of another person. Apart from ideas, thoughts, or fantasies, one can also experience fear, shame, and embarrassment [9]. All three dimensions should be taken into account when considering the concept of sexuality. Only a holistic approach to sexuality will enable a full understanding of its complexity [10].

The problem of urinary incontinence affects the patient’s daily life, significantly reducing the health-related quality of life, including the quality of sex life. Patients with incontinence often feel discomfort during daily activities such as: Physical activity or coughing, have low self-esteem, and depressed mood. The quality of personal, social, and professional life is destabilized. Women force themselves to change their lifestyle—they limit social contacts, which causes lower self-esteem and social isolation. Human alienation can lead to depression and anxiety disorders [11,12].

In order to cover up their embarrassing ailments, women change their sexual activity. Women are afraid that their partner will discover their intimate problem, so they consciously give up sexual activity. Sexual abstinence can lead to a loosening of the psychological bond of both partners. From the gynecological/sexological interview, women with urinary incontinence are concerned about the involuntary leakage of urine during sex. Patients are upset that their partner smells unpleasant urine or notices stained underwear [13,14].

Urinary incontinence can lead to major problems that negatively affect a woman’s sex life [15,16]. They are caused by certain processes taking place in the body during intercourse. As a result of an orgasm, intra-abdominal pressure increases, which in women with urinary incontinence can cause uncontrolled leakage of urine. The resulting discomfort, in turn, lowers sexual attractiveness and causes embarrassment [17]. Such experiences result in decreased libido, reluctance to engage in sexual intercourse, and orgasmic dysfunction [18,19].

Several researchers have analyzed the relationship between sexuality and various types of urinary incontinence. Asoglu [20] stated in his study that symptoms related to mixed urinary incontinence are associated with anxiety disorders, low-quality sexual life, and mood disorders. All those factors contribute to the occurrence of depression. On the other hand, Su [21] attempted to assess sexual dysfunction in patients with urinary incontinence using the international Female Sexual Function Index (FSFI) questionnaire. The research results revealed that affected domains of sexual function depend on the type of urinary incontinence. Urge urinary incontinence leads to vaginal dryness and pain during sexual activity. A mixed type of urinary incontinence reduces sexual satisfaction. According to the researcher, stress urinary incontinence does not affect sexual relations. However, little research into the effects of surgical treatment on urinary incontinence in terms of sexuality has been conducted. One of the few findings is that the procedures with the use of the midurethral slings may influence the bias towards sexual activity. It has also been recognized that patients with UI also suffer from dyspareunia and decreased libido, and they experience a complete cessation of sexual activity [22].

The study aims to evaluate the effect of surgical treatment of transobturator tape (TOT) procedure on the sexuality of women with stress urinary incontinence.

## 2. Materials and Methods

### 2.1. Design and Data Collection

The study involved 50 patients hospitalized in the period from February to May 2019 at the Gynecology Department of the Independent Public Healthcare Center of the Ministry of the Interior and Administration in Wrocław. All patients underwent surgical treatment of stress urinary incontinence using the TOT method. The study was approved by the Bioethics Committee of the Medical University of Wrocław with the number KB- 806/2018.

To assess sexuality, the international standardized Female Sexual Function Index (FSFI) questionnaire was used. The questionnaires were distributed to the patient on admission to the ward before the TOT procedure and during the follow-up visit, which took place 6 months after the procedure. The questionnaires were written by the patients in private space. The patients gave informed written consent to participate in the study. Patients were qualified for the study on the basis of the inclusion and exclusion criteria.

The inclusion criteria were as follows:

- Stage II and stage III stress incontinence confirmed by ultrasound scan and interview;
- hormone replacement therapy (HRT) before or after the surgical procedure;
- informed written consent of the patient to take part in the project;
- age: 45–65 years.

The exclusion criteria were as follows:

- women with overactive bladders (OAB) or mixed urinary incontinence (MUI);
- women with urinary tract fistulas;
- women with congenital or acquired defects of the urethra or bladder;
- women with urinary tract infections;
- women taking medicines contributing to an overactive bladder.

All the female patients selected for the project had the procedure of inserting the midurethral tapes using the transobturator (TOT) method. The treatments were performed by one surgeon. ABBIS CYRENE slings were used for them. The principles during the surgery included the following procedures:

- Inserting a catheter in the urinary bladder;
- incision and dissection of the vaginal mucosa and fascia;
- proper insertion of the tape;
- preventing implants from wrapping and rolling up;
- avoiding infection of the implants;
- optimal tension-free stitching of vaginal walls [23].

In all patients, the TOT procedure was correct and ended with a positive result. After surgery, symptoms of stress urinary incontinence decreased as assessed by interview and physical examination. The TOT method is now considered by many authors as one of the most effective methods of SUI treatment [24].

### 2.2. Measures

The Female Sexual Function Index includes questions that concern the basic emotional and physiological areas related to sexual contacts during the previous 4 weeks. It consists of 19 multiple-choice questions with increasing scores ranging from 0 to 5 regarding the presence of the questioned function, and 6 domains: Desire (questions 1 and 2), arousal (questions 3–6), lubrication (questions 7–10), experiencing orgasm (questions 11–13), satisfaction with the emotional and sexual relationship with a partner (questions 14 –16), and pain during intercourse (questions 17–19). Individual responses are assigned point values, which make up the total score on a scale from 2.0 to 36.0, with values ≤ 26 indicating sexual dysfunction [25].

## 3. Statistical Analysis

Statistical analysis was performed using the R Project. Descriptive statistics analysis carried out by means of the Wilcoxon test and chi square test. Quantitative variables were presented using the arithmetic mean, median, standard deviation, and minimum and maximum values. Statistical tests were carried out with a significance level of *p* = 0.05.

## 4. Results

The project involved 50 women with a diagnosis of stage II and stage III stress incontinence. The mean age of the patients was 57.2 years, and the range was 45–60 years. The mean age of women is 57.2 years (median 57.5). The youngest person was 45, and the oldest 65. Only 18% were women under 50. The most numerous group were women aged 51–60 (44%). Patients aged 61–65 accounted for 38% of the entire study group.

**Figure 1:**
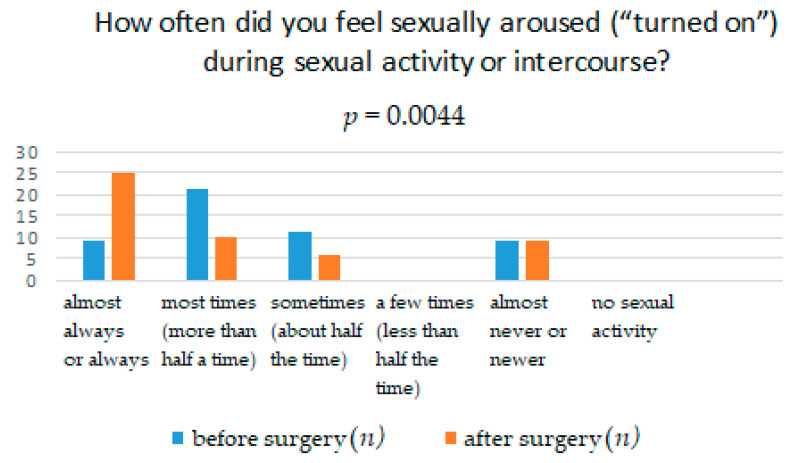
Feeling sexually aroused (“turned on”) during sexual activity or intercourse.

**Figure 2:**
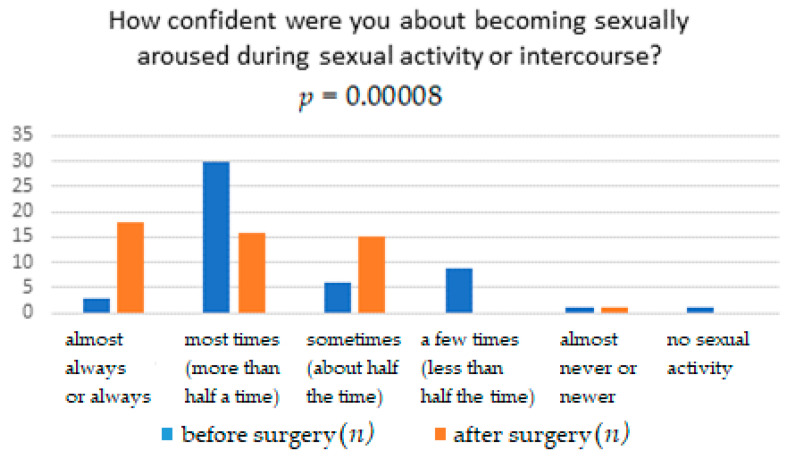
Confidence about becoming sexually aroused during sexual activity or intercourse.

**Figure 3:**
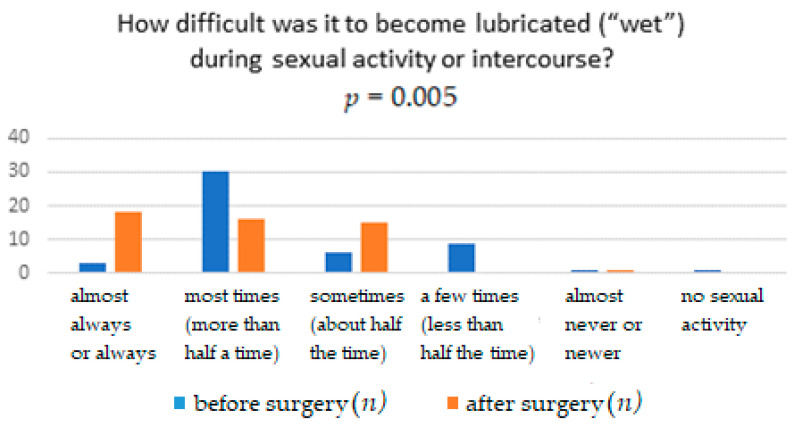
Difficulty to lubricated (“wet”) during sexual activity or intercourse.

**Figure 4:**
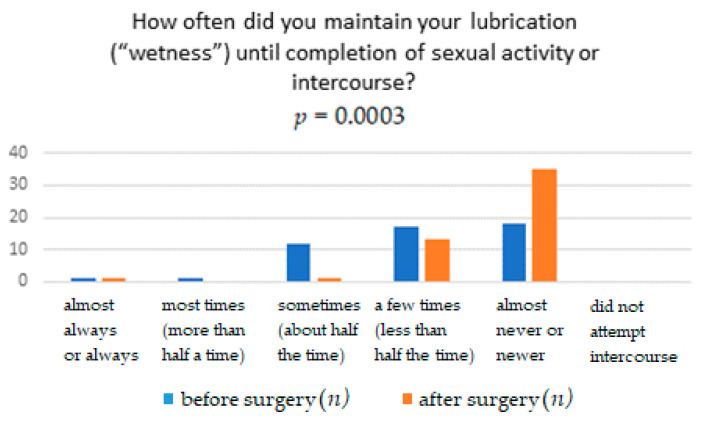
Frequency of lubrication (“wetness”) until completion of sexual activity or intercourse.

**Figure 5:**
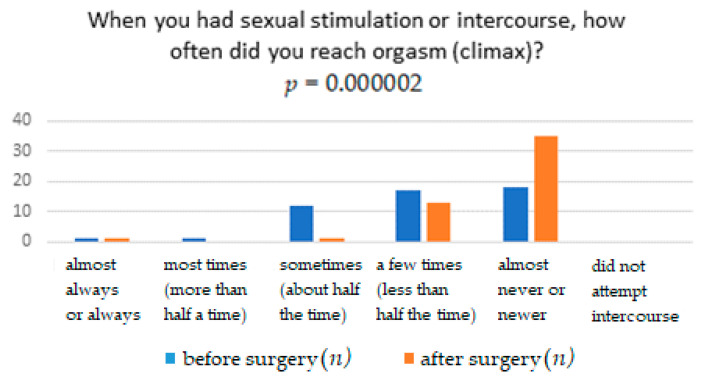
Reach orgasm (climax).

**Figure 6:**
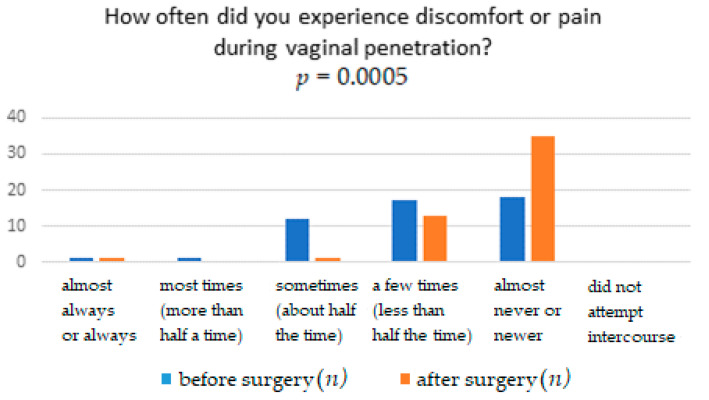
Frequency of feeling discomfort or pain during vaginal penetration.

The results obtained from the FSFI questionnaire show that surgery significantly affects the improvement in terms of pain, with *p* = 0.0041. It means that women after the TOT midurethral sling have less pain during sexual intercourse than women who have not undergone such treatment (Table 1). The distribution of responses to individual questions of the questionnaire is presented in Table 2, Table 3, Table 4, Table 5, Table 6 and Table 7. Figure 1, Figure 2, Figure 3, Figure 4, Figure 5 and Figure 6 show the results with statistically significant differences.

The relationship between surgery and the sex drive and sexual desire experienced by patient was also evaluated. The differences before and after surgery are small and statistically insignificant. Only 2% of women declare that they almost always or always felt sexual interest in the same way before and after the procedure. On the other hand, the level of sexual desire was evaluated on the same level both before and after the surgery (Table 2).

Analyzing the relationship in terms of being sexually aroused (“turned on”) during sexual activity in surgically treated women, it was noticed that the sexual arousal they felt was greater than before the surgery (Figure 1). Table also shows the relationship between surgical treatment of urinary incontinence and its impact on the awareness of women’s increasing excitement during sexual activity (Figure 2). After surgical treatment, patients are more often convinced of increasing excitement during sexual activity than before treatment (Table 3).

The relationship between surgery and difficulties in achieving vaginal lubrication during sexual activity was also analyzed. The result of the analysis turned out to be statistically significant (Figure 3). Moreover, the relationship between the ability to maintain vaginal lubrication and the surgery treatment conducted was evaluated (Figure 4). The result of the analysis turned out to be statistically significant (Table 4).

When analyzing the relationship between the occurrence of orgasm during sexual stimulation and the operation of stress urinary incontinence, significant statistical differences were noted (Table 5). After the procedure, the number of women who had an orgasm always or almost always increased (Figure 5).

Among the surveyed patients, the level of satisfaction with their sexual relationship with a partner is the same as compared to before and after surgery. In the study group, the general sexual life of women before the operation does not differ from the situation after the operation. Surgical treatment of UI does not significantly affect the satisfaction of patients with the amount of emotional closeness received from the partner during sexual activity (Table 6).

The relationship between the occurrence of pain and discomfort during sexual intercourse in women before and after surgery was also assessed (Figure 6). Postoperative women experience less discomfort and pain during sexual penetration (Table 7). 

The relationship between surgeries and difficulties in achieving vaginal lubrication during sexual activity was also analyzed. The result of the analysis turned out to be statistically significant (*p* < 0.05).

Moreover, the relationship between the ability to maintain vaginal lubrication and the surgical treatment was evaluated. The result of the analysis turned out to be statistically significant. 

## 5. Discussion

Until the 1960s, sexuality was a taboo subject, associated with procreation only. The situation changed when the role of women in society increased, and along with it, social changes took place. The burgeoning healthcare system has revealed an embarrassing problem concerning sexual dysfunction in many women. It has been shown that the problem most often affects menopausal women, especially those with urinary incontinence. Research conducted over the years has confirmed that incontinence affects the mental sphere, and in particular the sexuality of patients. The problem causes a feeling of stigmatization in the society, contributes to the occurrence of depression and anxiety, and also worsens the relationship with the partner [26].

The largest study on this problem, by Rogers et al. [27], involved 269 women. For the assessment of sexual function, PISQ and IIQ-7 questionnaires were used. They were completed by patients before treatment, and 3 months and 6 months after the surgery. This study did not address the influence of the procedure on the frequency of intercourse, willingness to have intercourse, nor the degree of excitement and satisfaction felt by patients and their partners. However, there was a statistically significant difference in the number of women achieving orgasm after surgery, which decreased from 32% to 17%. 

Similar results were presented by Caruso et al. [28], who published a study on changes in blood flow to the clitoris procedural access obturator, compared to treatments using access transmitting. The study involved 105 women, including 42 with TVT and 63 with TOT. The degree of blood supply to the clitoral area was examined through a transperineal ultrasound using color Doppler (before the procedure and 6 months after the procedure), assessing the maximum systolic flow, resistance index, and pulsation index. 88.2% of women in the TVT group and 95% in the TOT group were cured of SUI. A statistically significant difference (*p* < 0.05) was revealed in clitoral blood supply between patients who underwent TVT compared to TOT, in favor of TOT. According to the researchers, a lack of blood circulation disorders may have a major impact on the non-occurrence of complications in the form of sexual disorders in women treated for SUI.

Another study, which involved 113 women, was carried out by Jha et al. [29]. It aimed to assess the sexual function of women before and after surgery for SUI. 80% of the patients underwent the TVT surgery, and 20% of patients were subjected to TOT surgery. Two questionnaires were used to evaluate women’s sexual function: ICIQ-SF and PISQ. The analysis of the questionnaires showed a significant improvement in the physical and partner related aspect of women’s sexual life, while the sexual behavior and emotional aspects related to intercourse remained at the preoperative level. It should be mentioned, however, that as many as 7 women did not engage in sexual intercourse 6 months after the surgery. The surgical technique used did not influence these results. The study also assessed the incidence of climacturia, which decreased significantly after the tape treatment (*p* < 0.002).

Another study of the influence of TVT on sex life, published by Mazouni et al. [30], shows that postural sling adversely affects sexual comfort in 25.6% of women. Only one patient in the study group reported a significant improvement resulting from the disappearance of climacturia, and 1/3 of patients reported a different feeling of sexual pleasure, probably caused by changes in the innervation of the operated area. Similar observations concerning climacturia were presented Ghezzi et al. [31] in the six-month follow-up after the surgery virtually all women, who indicated the improvement of sexual life, belong to the group, which eliminated climacturia. At the same time, the procedure did not affect the occurrence of dyspareunia, which appeared de novo only in the patient with an erosion of the tape. In the study by El-Azab et al. [32] based on a letter questionnaire containing 9 questions concerning sexual life, the conclusions are similar. As many as 96 out of 128 women replied to the letter, 69 of whom were sexually active. 26% of respondents reported an improvement in their sexual pleasure, but on the other hand, 25% of them reported de novo discomfort felt by their partner due to vaginal narrowing or decreased vaginal lubrication.

In the presented study, the number of patients who experienced sexual arousal more often after the surgery increased, and their lubrication increased. However, there was no statistically significant change in terms of sexual satisfaction.

One of the causes of sexual dysfunction in women with urinary incontinence is pain. Dyspareunia is a specific type of pain associated with sexual activity. It is caused by urological disorders. In this research group, the occurrence of pain during sexual activity was observed. It should be emphasized that during stress urinary incontinence surgery, the number of women experiencing pain decreased statistically significantly. The results of the studies by Lemack et al. [33] are consistent with those of the present study and indicate that 20% of women surgically treated for SUI reported pain during penetration after one year. As compared to the state before surgery, this represents a slightly lower percentage of cases (29%).

In this study, after surgery, statistically significantly fewer patients experience pain during sex. The number of patients who never or almost never experience pain has increased by 34%.

Many authors reported that sexual function of women who had undergone surgical treatment for urinary incontinence is not significantly improved even despite the reduction in symptoms of urinary incontinence [34,35,36,37]. Numerous researchers mention in their publications that the treatment of urinary incontinence causes disorders related to orgasm in women, loss of libido, and decreased satisfaction with sexual intercourse [38,39,40,41,42,43]. In the presented study, no improvement in the degree of desire was observed in patients when comparing the status before and after surgery. However, the patients admitted that after surgery, they experienced greater sexual arousal during sexual activity. According to the publication by Simonelli [44], as many as 30.5% of the surveyed women report a decrease in sexual activity caused by fear of orgasm and the symptoms of “losing” urine that may accompany it.

In the present study, the number of women experiencing orgasm increased statistically significantly. The number of women who have experienced an orgasm has always or almost always increased by 30%.

Reviewing the published studies, it cannot be clearly stated that the surgical treatment of urinary incontinence improves sexual function of women. On the other hand, it should be taken into account that the lack of changes in some emotional and sexual behaviors, e.g., the degree of sexual satisfaction or the frequency of intercourse, depends not only the patients, but also on her relationship with their sexual partner. Based on the presented data, it cannot be unequivocally stated that the surgical procedure for SUI is devoid of side effects and does not significantly affect women’s sexual life. In some cases, surgical treatment for SUI is the only effective tool to restore sexual activity.

Summing up these preliminary results of the impact of surgical treatment on women’s sexual life, it should be considered promising, nevertheless establishing precise criteria and diagnostic tools seems necessary and requires a lot of research and establishing clear criteria and definitions to be able to fully meet the growing requirements of a modern woman. 

The results of these studies were also intended to show the importance of comprehensive care for a patient with stress urinary incontinence. The patient’s mental state should be assessed both before and after surgery. This research shows that a lot of attention should also be paid to issues related to sexuality. Operations performed with the TOT method have a significant impact on the improvement of sexuality in patients, which is why they are justified in this respect as well.

## 6. Conclusions

Stress urinary incontinence can significantly affect the sexual life of women.

Surgical treatment for stress urinary incontinence in the studied patients resulted in more frequent orgasms, fewer patients experienced pain and discomfort during intercourse, and lubrication during intercourse also improved.

The causes of female sexual dysfunction are complex, as they may result not only from urological problems, but also from the quality of women’s relationship with their partner.

## Figures and Tables

**Table 1:** Domain scores of the Female Sexual Function Index (FSFI) questionnaire from women before and after surgery.

	Before Surgery *n* = 50	After Surgery *n* = 50	*p*-Value
**Desire**	57.14 (0–100) 49.43 (27.11)	57.14 (0–100) 52.57 (27.30)	0.3104
**Arousal**	67.86 (0–100) 68.86 (24.18)	85.71 (14.29–100) 73.86 (20.49)	0.5960
**Lubrication**	95.00 (0–100) 85.90 (20.72)	95.00 (20–100) 83.90 (17.62)	0.2294
**Orgasm**	86.67 (0–100) 79.20 (17.80)	86.67 (20–100) 83.60 (13.88)	0.1181
**Satisfaction**	75.00 (0–100) 72.50 (22.03)	75.00 (0–100) 75.67 (18.04)	0.8808
**Pain**	63.64 (0–100) 69.09 (16.22)	63.64 (45.45–100) 64.18 (7.66)	0.0041

Data are presented as median (range) and mean (standard deviation); given *p*-values are for the Wilcoxon test for dependent samples.

**Table 2:** Desire domain responses from FSFI questionnaire from women before and after surgery.

Question	Response	Before Surgery *n* (%)	After Surgery *n* (%)	*p*-Value
**How often did you feel sexual desire or interest?**	Almost always or always	1 (2%)	1 (2%)	0.3022
	Most times (more than half a time)	11 (22%)	19 (38%)	
	Sometimes (about half the time)	26 (52%)	21 (42%)	
	A few times (less than half the time)	2 (4%)	0 (0%)	
	Almost never or never	10 (20%)	9 (18%)	
**How would you rate your level (degree) of sexual desire or interest?**	Very high	0 (0%)	0 (0%)	1
	High	5 (10%)	4 (8%)	
	Moderate	31 (62%)	32 (64%)	
	Low	5 (10%)	5 (10%)	
	Very low or none at all	9 (18%)	9 (18%)	

Data are presented as subgroups size. Given *p*-values are for the Fisher’s exact test.

**Table 3:** Arousal domain responses from FSFI questionnaire from women before and after surgery.

Question	Response	Before Surgery *n* (%)	After Surgery *n* (%)	*p*-Value
**How often did you feel sexually aroused (“turned on”) during sexual activity or intercourse?**	Almost always or always	9 (18%)	25 (50%)	0.0044
	Most times (more than half a time)	21 (42%)	10 (20%)	
	Sometimes (about half the time)	11 (22%)	6 (12%)	
	A few times (less than half the time)	0 (0%)	0 (0%)	
	Almost never or never	9 (18%)	9 (18%)	
	No sexual activity	0 (0%)	0 (0%)	
**How would you rate your level of sexual arousal (“turn on”) during sexual activity or intercourse?**	Very high	1 (2%)	2 (4%)	0.8181
	High	19 (36%)	22 (44%)	
	Moderate	13 (26%)	15 (30%)	
	Low	15 (30%)	10 (20%)	
	Very low or none at all	1 (2%)	1 (2%)	
	No sexual activity	1 (2%)	0 (0%)	
**How confident were you about becoming sexually aroused during sexual activity or intercourse?**	Very high confidence	17 (34%)	7 (14%)	0.00008
	High confidence	11(22%)	30 (60%)	
	Moderate confidence	7 (14%)	10 (20%)	
	Low confidence	13 (26%)	2 (4%)	
	Very low or no confidence	1 (2%)	1 (2%)	
	No sexual activity	1 (2%)	0 (0%)	
**How often have you been satisfied with your arousal (excitement) during sexual activity or intercourse**?	Almost always or always	17 (34%)	14 (28%)	0.3561
	Most times (more than half a time)	19 (38%)	28 (56%)	
	Sometimes (about half the time)	11 (22%)	7 (14%)	
	A few times (less than half the time)	1 (2%)	0 (0%)	
	Almost never or never	1 (2%)	1 (2%)	
	No sexual activity	1(2%)	0 (0%)	

Data are presented as subgroups size. Given *p*-values are for the Fisher’s exact test.

**Table 4:** Lubrication domain responses from FSFI questionnaire from women before and after surgery.

Question	Response	Before Surgery *n* (%)	After Surgery *n* (%)	*p*-Value
**How often did you become lubricated (“wet”) during sexual activity or intercourse?**	Almost always or always	21 (42%)	27 (52%)	0.4697
	Most times (more than half a time)	12 (24%)	14 (28%)	
	Sometimes (about half the time)	14 (28%)	7 (14%)	
	A few times (less than half the time)	1 (2%)	1 (2%)	
	Almost never or never	1 (2%)	1 (2%)	
	No sexual activity	1 (2%)	0 (0%)	
**How difficult was it to become lubricated (“wet”) during sexual activity or intercourse?**	Extremely difficult or impossible	1 (2%)	1 (2%)	0.0050
	Very difficult	0 (0%)	8 (16%)	
	Difficult	4 (8%)	0 (0%)	
	Slightly difficult	7 (14%)	7 (14%)	
	Not difficult	37 (72%)	34 (68%)	
	No sexual activity	1 (2%)	0 (0%)	
**How often did you maintain your lubrication (“wetness”) until completion of sexual activity or intercourse?**	Almost always or always	32 (64%)	15 (30%)	0.0003
	Most times (more than half a time)	7 (14%)	18 (36%)	
	Sometimes (about half the time)	6 (12%)	3 (6%)	
	A few times (less than half the time)	2 (4%)	12 (24%)	
	Almost never or never	2 (4%)	2 (4%)	
	No sexual activity	1 (2%)	0 (0%)	
**How difficult was it to maintain your lubrication (“wetness”) until completion of sexual activity or intercourse?**	Extremely difficult or impossible	2 (4%)	4 (8%)	0.3389
	Very difficult	0 (0%)	0 (0%)	
	Difficult	3 (6%)	0 (0%)	
	Slightly difficult	8 (16%)	7 (14%)	
	Not difficult	36 (72%)	39 (78%)	
	No sexual activity	1 (2%)	0 (0%)	

Data are presented as subgroups size. Given *p*-values are for the Fisher’s exact test.

**Table 5:** Orgasm domain responses from FSFI questionnaire from women before and after surgery.

Question	Response	Before Surgery *n* (%)	After Surgery *n* (%)	*p*-Value
**When you had sexual stimulation or intercourse, how often did you reach orgasm (climax)?**	Almost always or always	3 (6%)	18 (36%)	0.000002
	Most times (more than half a time)	30 (60%)	16 (32%)	
	Sometimes (about half the time)	6 (12%)	15 (30%)	
	A few times (less than half the time)	9 (18%)	0 (0%)	
	Almost never or never	1 (2%)	1 (2%)	
	No sexual activity	1 (2%)	0 (0%)	
**When you had sexual stimulation or intercourse, how difficult was it for you to reach orgasm (climax)?**	Extremely difficult or impossible	1 (2%)	1 (2%)	0.9288
	Very difficult	0 (0%)	0 (0%)	
	Difficult	2 (4%)	2 (4%)	
	Slightly difficult	10 (20%)	13 (26%)	
	Not difficult	36 (72%)	34 (68%)	
	No sexual activity	1 (2%)	0 (0%)	
**How satisfied were you with your ability to reach orgasm (climax) during sexual activity or intercourse?**	Very satisfied	10 (20%)	6 (12%)	0.7165
	Moderately satisfied	32 (64%)	39 (78%)	
	About equally satisfied and dissatisfied	4 (8%)	3 (6%)	
	Moderately dissatisfied	2 (4%)	1 (2%)	
	Very dissatisfied	1 (2%)	1 (2%)	
	No sexual activity	1 (2%)	0 (0%)	

Data are presented as subgroups size. Given *p*-values are for the Fisher’s exact test.

**Table 6:** Satisfaction domain responses from FSFI questionnaire from women before and after surgery.

Question	Response	Before Surgery *n* (%)	After Surgery *n* (%)	*p*-Value
**How satisfied have you been with the amount of emotional closeness during sexual activity between you and your partner?**	Very satisfied	11 (22%)	17 (34%)	0.2427
	Moderately satisfied	28 (56%)	19 (38%)	
	About equally satisfied and dissatisfied	9 (18%)	13 (26%)	
	Moderately dissatisfied	0 (0%)	0 (0%)	
	Very dissatisfied	1 (2%)	1 (2%)	
	No sexual activity	1 (2%)	0 (0%)	
**How satisfied have you been with your sexual relationship with your partner?**	Very satisfied	12 (24%)	17 (34%)	0.6301
	Moderately satisfied	27 (54%)	26 (52%)	
	About equally satisfied and dissatisfied	6 (12%)	5 (10%)	
	Moderately dissatisfied	4 (8%)	1 (2%)	
	Very dissatisfied	1 (2%)	1 (2%)	
	No sexual activity	0 (0%)	0 (0%)	
**How satisfied have you been with your overall sexual life?**	Very satisfied	11 (22%)	5 (10%)	0.1893
	Moderately satisfied	29 (58%)	39 (78%)	
	About equally satisfied and dissatisfied	5 (10%)	4 (8%)	
	Moderately dissatisfied	4 (8%)	1 (2%)	
	Very dissatisfied	1 (2%)	1 (2%)	
	No sexual activity	0 (0%)	0 (0%)	

Data are presented as subgroups size. Given *p*-values are for the Fisher’s exact test.

**Table 7:** Pain domain responses from FSFI questionnaire from women before and after surgery.

Question	Response	Before Surgery *n* (%)	After Surgery *n* (%)	*p*-Value
**How often did you experience discomfort or pain during vaginal penetration?**	Almost always or always	1 (2%)	1 (2%)	0.0005
	Most times (more than half a time)	1 (2%)	0 (0%)	
	Sometimes (about half the time)	12 (24%)	1 (2%)	
	A few times (less than half the time)	17 (34%)	13 (26%)	
	Almost never or never	18 (36%)	35 (70%)	
	Did not attempt intercourse	0 (0%)	0 (0%)	
**How often did you experience discomfort or pain following vaginal penetration?**	Almost always or always	1 (2%)	1 (2%)	0.2234
	Most times (more than half a time)	1 (2%)	1 (2%)	
	Sometimes (about half the time)	3 (6%)	9 (18%)	
	A few times (less than half the time)	13 (26%)	7 (14%)	
	Almost never or never	31 (62%)	32 (64%)	
	Did not attempt intercourse	1 (2%)	0 (0%)	
**How would you rate your level (degree) of discomfort or pain during or following vaginal penetration?**	Very high	1 (2%)	1 (2%)	0.4481
	High	0 (0%)	0 (0%)	
	Moderate	9 (18%)	16 (32%)	
	Low	15 (30%)	12 (24%)	
	Very low or none at all	24 (48%)	21 (42%)	
	Did not attempt intercourse	1 (2%)	0 (0%)	

Data are presented as subgroups size. Given *p*-values are for the Fisher’s exact test.

## Data Availability

The datasets used and/or analyzed during the current study are available from the corresponding author on reasonable request.
